# The Association of Familial Hypertension and Risk of Gestational Hypertension and Preeclampsia

**DOI:** 10.3390/ijerph18137045

**Published:** 2021-07-01

**Authors:** Małgorzata Lewandowska

**Affiliations:** 1 Medical Faculty, Lazarski University, 02-662 Warsaw, Poland; mal2015lewandowska@gmail.com; 2 Division of Gynecological Surgery, University Hospital, 60-535 Poznan, Poland

**Keywords:** preeclampsia, gestational hypertension, family history, paternal hypertension, maternal hypertension, obesity, smoking

## Abstract

It has not been established how history of hypertension in the father or mother of pregnant women, combined with obesity or smoking, affects the risk of main forms of pregnancy-induced hypertension. A cohort of 912 pregnant women, recruited in the first trimester, was assessed; 113 (12.4%) women developed gestational hypertension (GH), 24 (2.6%) developed preeclampsia (PE) and 775 women remained normotensive (a control group). Multiple logistic regression was used to calculate adjusted odds ratios (AOR) (and 95% confidence intervals) of GH and PE for chronic hypertension in the father or mother of pregnant women. Some differences were discovered. (1) Paternal hypertension (vs. absence of hypertension in the family) was an independent risk factor for GH (AOR-a = 1.98 (1.2–3.28), *p* = 0.008). This odds ratio increased in pregnant women who smoked in the first trimester (AOR-a = 4.71 (1.01–21.96); *p* = 0.048) or smoked before pregnancy (AOR-a = 3.15 (1.16–8.54); *p* = 0.024), or had pre-pregnancy overweight (AOR-a = 2.67 (1.02–7.02); *p* = 0.046). (2) Maternal hypertension (vs. absence of hypertension in the family) was an independent risk factor for preeclampsia (PE) (AOR-a = 3.26 (1.3–8.16); *p* = 0.012). This odds ratio increased in the obese women (AOR-a = 6.51 (1.05–40.25); *p* = 0.044) and (paradoxically) in women who had never smoked (AOR-a = 5.31 (1.91–14.8); *p* = 0.001). Conclusions: Chronic hypertension in the father or mother affected the risk of preeclampsia and gestational hypertension in different ways. Modifiable factors (overweight/obesity and smoking) may exacerbate the relationships in question, however, paradoxically, beneficial effects of smoking for preeclampsia risk are also possible. Importantly, paternal and maternal hypertension were not independent risk factors for GH/PE in a subgroup of women with normal body mass index (BMI).

## 1. Introduction

Pregnancy-induced hypertension (PIH) is a serious public health problem [1]. This disease, which is characteristic of pregnancy, develops de novo after 20 weeks of gestation and includes gestational hypertension (GH) and preeclampsia (PE) as well as PE superimposed on chronic hypertension [2]. PIH affects an average of 10% of pregnant women, although in some regions of the world this percentage is much higher [1,3,4]. Preeclampsia (PE) (in which high blood pressure is accompanied by particular organ disorders) occurs on average in 2–5% of pregnancies [2] but is seven times more common in developing than in developed countries [5]. Due to preeclampsia, 76,000 mothers and 0.5 million newborns die worldwide each year [2]. Pregnancy-induced hypertension is also a risk factor of cardiovascular disease and metabolic disorders later in life for both the mother and the child [2,3,6,7]. Early qualification of pregnant women with increased supervision (before pregnancy, or at its beginning) can promote the health of the mother and baby.

The recognized PIH risk factors include pre-pregnancy obesity/overweight, maternal age, primiparity, infertility treatment and smoking as well as preeclampsia in previous pregnancies and pre-existing hypertension. A family history of hypertension is also considered [2,7]. Family history of hypertension (which is already available before pregnancy) may predispose pregnant women to develop PIH, providing information about the potential influence of genetic and environmental factors [2,4,7,8,9,10].

However, the role of the family history of hypertension as an independent risk factor of pregnancy-induced hypertension (PIH) has not been clearly established [4,8,11,12,13,14,15,16,17,18]. Only the Society of Obstetricians and Gynaecologists of Canada (SOGC) 2014 guidelines list a family history of early cardiovascular disease among the risk factors for preeclampsia (PE) that determine the use of aspirin prophylaxis, in contrast to the guidelines of other scientific societies [11].

To date, only two studies assessed paternal or maternal hypertension separately as a risk factor of preeclampsia (PE), but the methodologies of these research were different [12,13]. In a systematic review published in 2021, it was not possible to perform a meta-analysis due to the lack of homogeneity of the studies [11]. Most of the studies to date have focused on preeclampsia risk (not on gestational hypertension) and are retrospective case-control studies [11].

To date, it has not been established how modifiable factors (such as pre-pregnancy body mass index (BMI) and smoking categories) affect the relationships in question. Can the family history of hypertension be an independent risk factor of pregnancy-induced hypertension also in the subgroup of pregnant women with normal BMI or who have never smoked?

The aim of this prospective study was to assess how chronic arterial hypertension in the father and (separately) mother of pregnant women in combination with maternal pre-pregnancy body mass index (BMI) categories or smoking categories affects the risk of gestational hypertension (GH) and (separately) preeclampsia (PE). No such study was found in the literature.

## 2. Materials and Methods

The data for this study come from a prospective cohort of women recruited at the Obstetrics and Gynecology Hospital of the Poznan University of Medical Sciences (Poland) in 2015–2016. This Research Center is a tertiary reference center for obstetrics, with 6000–8000 births annually.

### 2.1. Ethics

All the procedures related to this research project were in line with the Helsinki Declaration and were approved by the Bioethics Committee of the Poznan University of Medical Sciences, Poland (No. 769/15). Participation in this study was voluntary. All participants signed an informed consent statement prior to the commencement of the procedures.

### 2.2. Inclusion Criteria

The primary cohort included pregnant women enrolled at the end of the first tri-mester (according to appropriate criteria). In this cohort the pregnancy outcomes taken from medical records were assessed after the end of puerperium.

The inclusion criteria covered the following characteristics: Caucasian race and residence in the region (Wielkopolska), age of pregnant woman between 18–45 years (at conception), gestational age during recruitment at 10–14th week and singleton pregnancy, delivery of a child without defects at a gestational age of ≥25 weeks, and the lack of any preexisting diseases (except for disorders related to abnormal weight) including the following chronic diseases: hypertension, diabetes, inflammatory (and immune) diseases, neurological disorders, renal or hepatic dysfunction and coagulation disorders. Both multiparous and primiparous women were included in the study; in both cases, family history was recorded as well as the history of prior hypertension in pregnancy (in multiparous women).

### 2.3. Method

The recruitment process was carried out at the Central Laboratory. Information about the research project was available to all the women who reported for routine testing. The Main Personal Questionnaire was used to collect data at the recruitment stage (at the 10–14th gestational week). Information was collected on the mother’s characteristics such as age, weight before pregnancy (self-reported), blood pressure before pregnancy (self-reported), height, smoking, alcohol use, and the use of drugs before pregnancy and in the first trimester, as well as the use of vitamin supplementation micronutrient for pregnant women. The information on the course of pregnancy to date, obstetric and gynecological history, as well as socio-economic and demographic data was also collected.

Data on diseases in the family (including chronic diseases such as hypertension or diabetes and pregnancy-induced hypertension in the mother or sister) was collected as well, detailing diseases in the father, mother, sisters and brothers, as well as the grandmothers and grandfathers. Importantly, the women answered the questionnaire on their own (in the presence of midwives).

The second stage of the study (after the end of pregnancy and puerperium) was collecting the information on pregnancy outcomes and possible complications in the mother. The details regarding pregnancy results and other data related to the weight change in pregnancy and perinatal blood pressure values were taken from the medical records, and the information on family history (included in the medical records) was verified. An additional questionnaire completed after the 12th week of puerperium (by e-mail or telephone) was also used: it included the information on changes in puerperal blood pressure and some additional information, such as changes in smoking habits during pregnancy. All women reported refraining from the consumption of alcohol or other stimulants during pregnancy.

All 1300 women who volunteered for this study and met the admission criteria at the 10–14th week of pregnancy were invited to complete the questionnaire. After the second stage of the study, 388 women were excluded due to the following reasons: delivery at gestational age <25th week, newborn with congenital diseases, thromboembolism or severe infections in pregnancy, hypertension diagnosed before the 20th week, diabetes mellitus diagnosed before the 18th week, lack of cooperation (48 women), as well as missing data (340 women). Finally, 912 women were qualified.

In the cohort of 912 women, 775 women remained normotensive, and 113 (12.4%) developed gestational hypertension (GH) and 24 (2.6%) developed preeclampsia (PE) (11 cases beginning < 34th week and 13 cases ≥ 34th week). Both the controls and the cases had a history of earlier GH or PE (prior GH/PE).

The main aim of the current study was to evaluate the relationship between chronic hypertension in the father or (separately) the mother and the risk of GH or PE.

The minimum sample size was calculated using the formula for a single proportion (for 95% confidence intervals and α = 5%; Z = 1.962: critical value of normal distribution at α/2; ‘p’: sample proportion; ‘d’: margin of error):

(1) $$n=Z_{\frac{\alpha }{2}\;}^{2}\frac{p\cdot \left(1-p\right)}{d^{2}}$$

The minimum sample size was 866 for the error value of d = 0.02 (2%) and for the mean proportion *p* = 0.1 (10%), cited in the literature [3]. For the proportion *p* = 0.124 (12.4%) and the error d = 0.03 (3%), the minimum sample size was 465.

Our cohort (*n* = 912) was large enough to discover the associations of interest to us.

### 2.4. Definitions of Dependent Variables

In this study, the dependent variables were both major pregnancy-induced hyper-tension (PIH) phenotypes, investigated separately. PIH was defined as blood pressure (systolic and diastolic) ≥140/90 mmHg, developing de novo after the 20th gestational week (obtained in at least two measurements four hours apart, and measured with an oscillometric device in a sitting position). (1) Gestational hypertension (GH) was diagnosed when de novo high blood pressure was not accompanied by any abnormalities in vital organs. (2) Preeclampsia (PE) was diagnosed when this arterial hypertension was accompanied by de novo development of the following organ disorders: renal dysfunction and/or hepatic disorders and/or thrombocytopenia, visual and/or cerebral disorders or pulmonary edema (in this cohort, de novo hypertension and proteinuria ≥300 mg/24 h were found in all PE cases).

### 2.5. Independent Variables

The independent variables were categories of the family history of chronic hypertension. During recruitment, the participants of the study reported information about their family histories independently (in the questionnaire), by answering the question: ‘Who in the family suffers from these chronic diseases?’ The information about the family’s medical history was part of the mother’s characteristics (not the main focus of the study). After childbirth, the data from the questionnaires were positively verified with the data from the pregnant women’s medical records. The questionnaires provided information on the family history of chronic diseases in the father (*n* = 179), mother (*n* = 173), sister(s) (*n* = 6), brother(s) (*n* = 7), grandmother(s) (*n* = 52) and grandfather(s) (*n* = 25). The results refer to the number of pregnant women who reported hypertension in their relatives (sisters/brothers or grandmothers/grandparents). Information obtained from five pregnant women was classified as missing (*n* = 5) (as the information was inconclusive).

The main independent variable was chronic arterial hypertension in the pregnant women’s parents. This study evaluated the following six categories of chronic hypertension: (1) hypertension in the mother only, (2) hypertension in the father only, (3) hypertension in the mother or father, (4) hypertension in the mother and father simultaneously, (5) Absence of hypertension in the parents, and (6) Absence of hypertension in the family (in the parents and other relatives). The reference categories were: ‘Absence of hypertension in the family’ and ‘Absence of hypertension in the parents’.

### 2.6. Covariates

In this study, the following covariates (Table 1) (risk factors for pregnancy-induced hypertension) were assessed: maternal age, pre-pregnancy body mass index (BMI), parity, gestational weight gain (GWG), smoking, prior pregnancy-induced hypertension (GH/PE cases), and infertility treatment, as well as education level and financial status [2,7,19]. Among the smokers, ‘smoking in the first trimester’ was used as a confounding variable according to our earlier results (it was associated with a significantly higher GH and PE odds ratios) [20]. In this study, no information on passive (secondhand) smoking was available.

### 2.7. Statistical Analyses

Statistica software (Version 13, TIBCO, Palo Alto, CA, USA) was used for the analyses. Maternal characteristics including family history of chronic diseases were compared between pregnant women who developed pregnancy-induced hypertension (PIH) (case group) and women who remained normotensive (control group) and were described by the number (and percentage) or using mean values (and standard deviation, SD). The Shapiro-Wilk test was used to investigate the normality of the data distribution. Continuous variables were not normally distributed, and the Mann-Whitney U test was used for comparisons of these variables. We used the Pearson chi-square test (or Fisher exact test when Cochran assumption was not met) for comparisons of binomial variables. The *p*-value < 0.05 was assumed to be significant.

Multiple logistic regression was used to calculate adjusted odds ratios (AOR) (and 95% confidence intervals) of gestational hypertension (GH) and preeclampsia (PE) for chronic hypertension in the father or mother of pregnant women. Univariate logistic regression was used to calculate unadjusted odds ratios (OR). The reference categories (with OR/AOR = 1.00) were: (1) ‘Absence of chronic hypertension in the family’ (in the parents and other relatives); and (2) ‘Absence of chronic hypertension in the parents’. The Wald test was used to calculate *p*-value (*p* < 0.05 was assumed to be significant).

In multiple logistic regression, the results were corrected for the following confounding variables: maternal age, pre-pregnancy body mass index (BMI), primiparity, gestational weight gain (GWG) out of the range and smoking in the first trimester (model-a), plus prior hypertension in pregnancy and infertility treatment (model-b).

The whole cohort and the subgroups (of smoking and pre-pregnancy BMI categories) were investigated.

Adjusting the results (in multiple logistic regression) for small groups requires fewer confounders. In the current analysis, the sizes of the preeclampsia (PE) group and subgroups were small, so two adjustment models were used, with a smaller (model-a) and a larger (model-b) number of confounding variables. Importantly, some independent variables (categories of family history of hypertension) were statistically significantly associated with the risk of GH and PE (in these small groups and subgroups), which means that these results had sufficient statistical power.

## 3. Results

### 3.1. Basic Characteristics of the Partcipants

Table 2 shows the basic characteristics of women who developed pregnancy-induced hypertension (PIH) (cases) and women who remained normotensive (controls). Women developing PIH had a statistically significantly higher mean age and mean pre-pregnancy body mass index (BMI), and were statistically significantly more likely to smoke cigarettes in the first trimester (14.6% vs. 4.8%). In the group of cases there were also more primiparous women (47.5% vs. 41.0%) and more women treating infertility (7.1% vs. 3.7%). In the PIH group, a statistically significantly lower gestational age of newborns and lower birth weight, as well as an insignificantly higher percentage of women who developed gestational diabetes mellitus (GDM), was found.

In the PIH group (vs. normotensive group) there were statistically significantly more women who developed GH/PE in the previous pregnancy (Prior GH/PE) (11.0% vs. 0.5%), as well as women whose mothers or sisters developed GH/PE (4.4% vs. 0.5%). In the PIH group, a statistically significantly higher frequency of chronic hypertension in the parents was found. Cases of ‘no parental hypertension’ and ‘no family history of hypertension’ were statistically significantly more common in the normotensive group.

Table 3 shows the adjusted odds ratios of gestational hypertension (GH) and preeclampsia (PE) for the basic risk factors calculated in multiple logistic regression (in model-a). The set of data (number of cases/controls and crude odds ratios) is presented in the Supplementary File (Supplementary Table S1).

Some differences were found. The highest GH and PE odds ratios were obtained for prior GH/PE. The study participants’ older age (≥40 years) was associated with a higher risk of GH (compared to the age of 25–29 years), but the youngest age (18–24 years) was associated with higher PE odds ratios. Primiparity (vs. multiparity) was associated with higher odds ratios of GH and PE, however, only the GH score was statistically significant. Infertility treatment, in turn, was statistically significantly associated with higher PE risk.

Socio-economic factors (lower education level and lower financial status) were also associated with a higher risk of GH and PE.

Among modifiable factors, pre-pregnancy obesity strongly increased the risk of preeclampsia (PE), while overweight and obesity was a risk factor for gestational hypertension (GH). Smoking in the first trimester (compared to women who never smoked) was associated with higher odds ratios of GH and PE.

### 3.2. Odds Ratios of GH and PE for Chronic Hypertension in the Parents

Table 4 shows the adjusted odds ratios of gestational hypertension (GH) and preeclampsia (PE) for chronic hypertension in the parents, calculated in multiple logistic regression. The size of the preeclampsia (PE) group was small, so two adjustment models were used, with a smaller (model-a) and a larger (model-b) number of confounding variables; both models were successfully built and the results in both models were similar.

Some interesting differences were discovered. Paternal hypertension (compared to ‘Absence of hypertension in the family’) was associated with a higher and statistically significant adjusted risk of gestational hypertension (GH) (AOR-a = 1.98 (1.2–3.28); AOR-b = 1.88 (1.12–3.17)) and was an independent GH risk factor (it was independent from the influence of other risk factors).

On the contrary, maternal hypertension (vs. ‘Absence of hypertension in the family’) was associated with a higher and statistically significant adjusted risk of preeclampsia (PE) (AOR-a = 3.26 (1.3–8.16); AOR-b = 3.09 (1.2–7.98)) and was an independent PE risk factor. The PE odds ratio for maternal chronic hypertension was more than twice as high as for paternal hypertension (Table 4).

The same relationships were obtained when the reference category was ‘Absence of hypertension in the parents’ (Supplementary Table S2).

### 3.3. GH and PE Risk after Cohort Dissection into BMI and Smoking Categories

Table 5 shows the GH and PE odds ratios for parental chronic hypertension calculated by subgroups (smoking and pre-pregnancy BMI categories). The analyses were performed in multiple logistic regression. The sizes of the subgroups were smaller, therefore a correction model was used with a smaller (model-a) number of confounding variables.

In the whole cohort, paternal hypertension (vs. ’Absence of hypertension in the family’) was associated with a higher risk of gestational hypertension (GH) (AOR-a = 1.98 (1.2–3.28)). This odds ratio increased in pregnant women who smoked in the first trimester (AOR-a = 4.71 (1.01–21.96)) or smoked before pregnancy (AOR-a = 3.15 (1.16–8.54)) or were overweight (AOR-a = 2.67 (1.02–7.02)).

Importantly, GH odds ratios were statistically insignificant in the subgroup of normal BMI (AOR-a = 1.84 (0.92–3.67)) and women who had never smoked (AOR-a = 1.70 (0.94–3.08)).

In the whole cohort, maternal hypertension (vs. ‘Absence of hypertension in the family’) was associated with a higher risk of preeclampsia (PE) (AOR-a = 3.26 (1.3–8.16)). This odds ratio increased in the obese women (AOR-a = 6.51 (1.05–40.25)), and (importantly) was statistically insignificant in the subgroup of normal BMI (AOR-a = 1.40 (0.26–7.6)).

The odds ratio of PE for maternal hypertension (paradoxically) increased in women who had never smoked (AOR-a = 5.31 (1.91–14.8)) and was lower in the subgroup of smokers before pregnancy (AOR-a = 0.88 (0.07–11.38)).

**Figure 1:**
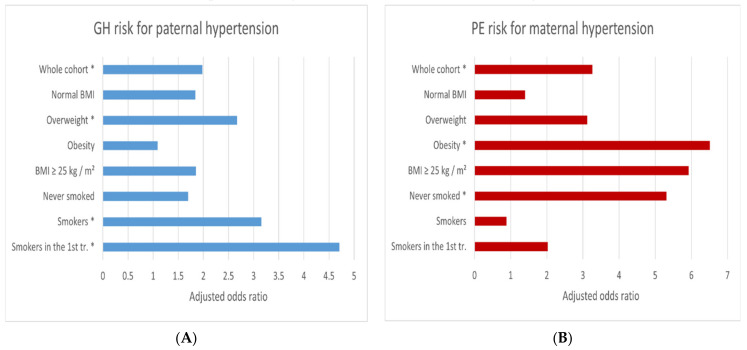
The adjusted odds ratios of gestational hypertension (GH) (**A**) and preeclampsia (PE) (**B**) for hypertension in the parents (vs. ‘Absence of hypertension in the family’) in the whole cohort and subgroups. The results were calculated in multiple logistic regression after adjusting for maternal age, pre-pregnancy body mass index (BMI), primiparity, gestational weight gain (GWG) out of the range and smoking in the first trimester (AOR-a). * Statistically significant results (Table 5).

Figure 1 shows the risk of gestational hypertension (GH) or preeclampsia (PE) for chronic parental hypertension following the division of the cohort into subgroups.

The graphs emphasize that the relationships of paternal hypertension with GH were statistically insignificant in women with normal BMI, but the risk increased in overweight women and among smokers (Figure 1A).

Relationships between maternal hypertension and PE were statistically insignificant in women with normal body mass index (BMI), but the risk increased in obese women and (paradoxically) in women who never smoked (Figure 1B).

## 4. Discussion

This study showed that chronic arterial hypertension in the parents of pregnant women affected the risk of preeclampsia (PE) and gestational hypertension (GH) in a different way. An independent risk factor for GH (independent from the influence of other confounders) was paternal hypertension, while an independent risk factor for PE was maternal hypertension.

GH odds ratio (for paternal hypertension) increased in smokers as well as pregnant women with pre-pregnancy overweight. Preeclampsia odds ratio (for maternal hypertension) increased sharply in obese pregnant women and (paradoxically) in those who never smoked. However, paternal and maternal hypertension were not independent risk factors for GH/PE in a subgroup of women with normal body mass index (BMI).

Some analyses in this study were performed in small subgroups, therefore caution is advised in their interpretation. However, statistically significant results were obtained despite the wider confidence intervals (CI), which suggests that these results are not random.

The clinical implications of this study include at least three aspects. (1) The family history of hypertension in the father or mother may be added to the risk factors of pregnancy-induced hypertension (PIH) that qualify women in early pregnancy to increased surveillance. This measure can promote the health of the mother and the baby. (2) The different role of ‘paternal’ and ‘maternal’ chronic hypertension may suggest genetic (and environmental) relationships. Additionally, the different role of the ‘paternal’ and ‘maternal’ hypertension we found may be the cause of discrepancies in the results when the ‘family history of hypertension’ is examined as a single variable. (3) Modifiable risk factors (overweight/obesity and smoking) may increase the relationships in question. The optimal pre-pregnancy weight of women in early pregnancy may protect against the emergence of the effect of genetic factors. However, paradoxically beneficial effects of smoking on preeclampsia risk are possible.

The present study adds some value to the existing literature: it was a prospective study (data were collected when pregnancy outcomes were unknown); the risk of gestational hypertension (GH) and preeclampsia (PE) was assessed separately in a joint cohort; the results were adjusted for the recognized risk factors of GH/PE; clearly presented definitions (of GH and PE, and hypertension in the father or mother) were used; the body mass index (BMI) and smoking subgroups were assessed separately.

To date, only three studies (from earlier years) assessed paternal and (separately) maternal chronic hypertension. In a case-control study of 190 women with preeclampsia and 373 healthy controls, Qiu et al., found statistically significantly higher adjusted risks of PE for maternal and paternal hypertension (AOR 1.9 and 1.8, respectively) [12]. Examining 162 primiparous women with severe preeclampsia and 521 healthy primiparous women, Rigo et al., also found statistically significant higher adjusted preeclampsia risk for maternal and paternal hypertension (AOR 3.84 and 3.26, respectively) [13]. Klonoff-Cohen et al., examined 110 nulliparous women who had preeclampsia and 115 healthy nulliparous; the authors found a higher frequency of maternal or paternal hypertension in the case group than in the control group [21]. In these studies, no difference was found between the role of maternal and paternal hypertension in the risk of preeclampsia (PE), which was contrary to our results (Table 4, Figure 1).

The studies to date have mainly focused on preeclampsia [11]. Twenty-six studies (out of 48) showed statistically significant associations of preeclampsia (PE) with ‘family history of hypertension’. Eight studies (out of 11) found associations of gestational hypertension (GH) with ‘family history of hypertension’ [11].

The discrepancies between the studies could be attributed to differences in clinical methodology, sizes of the studied groups, risk of the studied populations, the numbers of early-onset preeclampsia (PE) (<34th weeks) and late-onset PE cases, ethnic differences, as well as differences in the definitions of ‘family hypertension’ or ‘hypertension in pregnancy’ and the degree of adjustment [4,8,11,12,13,21].

This cohort study showed that the prevalence of pregnancy-induced hypertension (PIH) was 15%. This result is more than twice as high as the results in other Polish studies [22,23]. This may be so as the testing was performed in a highly referenced center where many women with risk factors report. However, in this study, the proportion of preeclampsia (PE) cases alone was 2.6%. This result is in line with the lower figures reported in the International Federation of Gynecology and Obstetrics (FIGO) report (2–5%) [2], but risk factors such as pre-existing hypertension, other chronic diseases and multiple pregnancies were excluded in this cohort.

Other characteristics of the participants related to the family history were also shown in this cohort. The incidence of gestational hypertension/preeclampsia (GH/PE) in the mother and sister (Table 2) was statistically significantly higher in pregnant women developing pregnancy-induced hypertension (PIH) than in pregnant women who remained normotensive, which is consistent with the literature [10]. In this study, we found no association between parental (paternal or maternal) diabetes and the GH or PE cases (Table 2), which is consistent with the results in the literature [11].

This study showed a large role of modifiable factors. Obesity is a known independent risk factor for pregnancy complications, including pregnancy-induced hypertension. In this study, the adverse effects of overweight/obesity were shown (Table 3). Cigarette smoke contains numerous toxic substances, but the effect of smoking on the risk of hypertension in pregnancy is inconclusive. The results of the current analysis showed the possibility of paradoxically beneficial effects of smoking in the development of preeclampsia (PE), as reported in the literature [24,25]. This is an interesting finding because in this cohort, smoking (as a basic risk factor) increased the preeclampsia (PE) and gestational hypertension (GH) odds ratios (Table 3), which was also examined in detail in our previous work [20]. The literature confirms that gestational hypertension (GH) and preeclampsia (PE) may have common and different risk factors [26,27,28].

Mechanisms for the relationship between the family history of chronic hypertension and the risk of GH and PE are not established. The etiology of GH/PE is not fully understood yet. Among many factors, genetic, metabolic, immunological and environmental factors are taken into consideration [2,7,29,30,31]. Excessive oxidative stress, inflammation and endothelial disorders are key elements of pathogenesis of GH and PE [2,7,32]. Numerous genes associated with preeclampsia have been identified, although the results are not conclusive [9,10,30,33,34,35].

Evidence of genetic susceptibility can be an increased risk of developing preeclampsia (PE) in women whose mothers also developed PE, an increased risk of PE in pregnant women with chronic hypertension, and an increased risk of cardiovascular disease in pregnant women who developed PE [2,7,8,10]. Maternal (genetic) factors may be associated with the etiology of early-onset PE, in whose development the importance of the primary pathology of the placental perfusion is emphasized [2,7,32]. Paternal factors may play a greater role in the etiology of gestational hypertension (GH) and late-onset preeclampsia (PE) (where the placenta is often normal), as their development suggests a greater importance of primary pre-pregnancy endothelial disorders (e.g., in women with overweight/obesity or hyperinsulinemia, or chronic hypertension before pregnancy) [2].

### Summary—Advantages and Limitations

The strength of this study was the prospective cohort study model (pregnancy outcomes were unknown at recruitment, and family history was reported prior to disease development). Another advantage is researching the paternal and maternal history of chronic hypertension separately. An advantage is the separate assessment of the risk of preeclampsia and gestational hypertension. In the risk assessment, we included many confounding variables, although there may be other factors that would influence the results. Additional analyses after stratification into subgroups of various smoking categories and pre-pregnancy body mass index (BMI) categories were also a strength of the study.

The participants of the study reported some anthropometric data, but the most important data came from the medical records. Family history (as well as smoking and pre-pregnancy weight) was reported by pregnant women in the questionnaires, however this information was also cross-checked with the information in the medical reports and/or in a supplementary questionnaire. No information on passive (secondhand) smoking was available.

In this study, the family history of chronic hypertension was established from a questionnaire interview. Details of the family history were not known (age threshold for developing chronic hypertension was unknown), which is a typical limitation of research on this topic [11].

A small number of preeclampsia cases is a limitation. The division into subgroups led to a decrease in the sample size, but many of the results were statistically significant despite the wider confidence intervals (characteristic of a small sample size). The age of pregnant women is important not only as a risk factor of pregnancy-induced hypertension; parents of younger women may have not yet developed hypertension, while on the other extreme are older women born to older mothers, but the results of this study were adjusted for maternal age, among other factors.

## 5. Conclusions

In this study, maternal chronic hypertension was an independent risk factor for preeclampsia (PE) (it was independent from other confounders), while paternal chronic hypertension was an independent risk factor for gestational hypertension (GH). Family history may be added to the risk factors of pregnancy-induced hypertension that qualify women in early pregnancy to the group of increased surveillance. This measure can promote the health of the mother and the baby.

This study also confirms the strong role played by modifiable risk factors (such as obesity or smoking) that can exacerbate the effects of genetic factors. Importantly, in this study, paternal and maternal hypertension were not independent risk factors for gestational hypertension and/or preeclampsia in a subgroup of women with normal body mass index (BMI).

The results of this analysis suggest that shared (family) lifestyle or susceptibility to obesity is likely to contribute to the tested effects. Improving the lifestyle before pregnancy (especially the normalization of BMI) may protect against the negative influence of genetic factors. However, the influence of smoking on the relationships in question requires further research in a larger sample.

## Figures and Tables

**Table 1:** Characteristics of the covariates and main dependent variables.

Variates	Definitions and Categories	Description
Pre-pregnancy BMI	(the quotient of pre-pregnancy weight (kg) and height (meters) squared) BMI was assessed as a continuous variable (as a covariate); BMI was assessed in the 5 following categories (as subgroups): (1) underweight (<18.5); (2) normal weight (18.5–24.9); (3) overweight (25.0–29.9); (4) obesity (≥30); and (5) BMI ≥ 25 kg/m^2^.	Self-reported
GWG	(the difference between the weight before childbirth and the weight before pregnancy) GWG was assessed in the three following categories, regardless of BMI category (according to the Institute of Medicine recommendations from 2009): (1) GWG above the range; (2) GWG in the range; (3) GWG below the range. GWG out of the range was a covariate.	From medical reports
Maternal age	(defined as completed maternal age at conception, in years) Maternal age was assessed as a continuous variable (as a covariate)	From medical reports
Primiparity	Parity was assessed in the two following categories: (1) primiparity (zero prior delivery); (2) multiparity (i.e., ≥1 prior deliveries). Primiparity was a covariate.	From medical reports
Smoking	Smoking was assessed in the three following categories: (1) mothers who had never smoked; (2) smokers (smoked before pregnancy); (3) smokers in the first trimester. Smokers in the first trimester was a covariate. The three smoking categories was assessed as subgroups.	Self-reported
Prior PIH	Mothers with pregnancy-induced hypertension (gestational hypertension or preeclampsia, GH/PE) in previous pregnancies. Prior PIH was assessed in the two following categories: (1) prior PIH; (2) no prior PIH. Prior PIH was a covariate.	From medical reports
Infertility treatment	(Different methods of infertility treatment, covering in vitro fertilization and other methods of assisting reproduction) Infertility treatment was assessed in the two following categories: (1) infertility treatment; (2) no infertility treatment. Infertility treatment was a covariate.	From medical reports
Education	Education level was assessed in the following categories: (1) ≥12 years of education (secondary education and tertiary education); (2) <12 years of education (primary and vocational education)	From medical reports
Financial status	Financial status was assessed on the 5-point Likert scale. The basis of assessment was the question ‘Is your household’s financial status sufficient for your needs?’ and the 5 following answers: ‘1—definitely No’; ‘2—rather No’; ‘3—hard to say’; ‘4—rather Yes’; ‘5—definitely Yes’. Financial status was assessed in the following categories: (1) Lower financial status (the answers/levels 1–3); and (2) Higher financial status (the answers/levels 4–5).	Self-reported

BMI—Body mass index; GWG—gestational weight gain; PIH—Pregnancy-induced hypertension.

**Table 2:** Basic characteristics of the women who developed pregnancy-induced hypertension (PIH).

Maternal Characteristics **	Normotensive Group (*n* = 775)	PIH Group (*n* = 137)	*p* *
	Mean (SD), *n* (%)	Mean (SD), *n* (%)	
Basic characteristics			
Maternal age (years)	33.5 (4.8)	34.8 (4.4)	0.005
Pre-pregnancy BMI (kg/m^2^)	23.3 (4.1)	26.7 (5.4)	<0.001
GWG (kg)	13.4 (5.3)	14.7 (8.0)	0.107
Primiparous women	318 (41.0%)	65 (47.5%)	0.161
Infertility treatment	29 (3.7%)	8 (7.1%)	0.097
Smokers in the 1st tr.	37 (4.8%)	20 (14.6%)	<0.001
Prior GH/PE	4 (0.5%)	15 (11.0%)	<0.001
GH/PE in the mother or sister	4 (0.5%)	6 (4.4%)	<0.001
Chronic diseases in family			
*Hypertension (H):*			
In the mother	137 (17.8%)	42 (30.9%)	<0.001
In the father	135 (17.5%)	38 (27.9%)	0.004
In the grandmother(s)	47 (6.1%)	5 (3.7%)	0.263
In the grandfather(s)	20 (2.6%)	5 (3.7%)	0.407
- Absence of H in the parents	541 (70.2%)	72 (52.9%)	<0.001
- Absence of H in the family	496 (64.3%)	69 (50.7%)	0.003
*Diabetes:*			
In the mother	55 (7.1%)	15 (11.0%)	0.117
In the father	90 (11.7%)	20 (14.7%)	0.318
Pregnancy outcomes			
Fetal sex—male	405 (52.3%)	68 (49.6%)	0.571
Gestational age (weeks)	38.8 (1.6)	37.7 (2.8)	<0.001
Birth weight (grams)	3416.5 (511.7)	3020.0 (838.0)	<0.001
GDM cases	121 (15.6%)	25 (18.3%)	0.438
SABP (mmHg) ***	107.9 (10.8)	159.5 (18.2)	<0.001
DABP (mmHg) ***	66.8 (8.8)	100.9 (11.0)	<0.001

* The Mann-Whitney U test was used for comparisons of continuous variables (the variables were not normally distributed) and the Pearson chi-square test was used (or Fisher’s exact test when Cochran assumption was not met) for comparisons of binomial variables (*p* < 0.05 was assumed to be significant); ** Analyses for available data; *** SABP: systolic arterial blood pressure; DABP: diastolic arterial blood pressure (measured after delivery). PIH, pregnancy-induced hypertension; BMI, body mass index; GWG, gestational weight gain; GDM, gestational diabetes mellitus.

**Table 3:** The adjusted odds ratios of two forms of pregnancy-induced hypertension for basic risk factors (model-a).

Basic Risk Factors	GH Risk	PE Risk
	AOR-a (95% CI); *p* *	AOR-a (95% CI); *p* *
*Pre-pregnancy BMI (kg/m^2^):*		
Obesity (≥30)	4.72 (2.73–8.14); <0.001	8.68 (3.30–23.20); <0.001
Overweight (25.0–29.9)	2.04 (1.23–3.37); 0.005	1.68 (0.50–5.63); 0.397
Underweight (<18.5)	0.23 (0.03–1.69); 0.148	2.56 (0.52–12.62); 0.248
Normal BMI (18.5–24.9)	1	1
Smoking in the 1st tr.	3.51 (1.79–6.89); <0.001	2.79 (0.76–10.27); 0.124
Smoking (ever)	1.64 (1.01–2.66); 0.044	0.88 (0.29–2.65); 0.817
Never smoked	1	1
GWG above the range	1.90 (1.15–3.12); 0.012	1.15 (0.44–3.00); 0.770
GWG below the range	1.09 (0.59–2.01); 0.779	0.84 (0.26–2.67); 0.768
GWG in the range	1	1
*Maternal age (years):*		
≥40	2.97 (1.2–7.32) 0.018	0.94 (0.08–11.6); 0.963
18–24	0.51 (0.1–2.53) 0.413	3.26 (0.42–25.0); 0.256
25–29	1	1
*Prior GH/PE*		
Yes	37.98 (11.16–129); <0.001	31.11 (5.83–166.0); <0.001
No	1	1
Primiparity	1.90 (1.22–2.96); 0.005	1.55 (0.64–3.77); 0.332
Multiparity	1	1
Education <12 years	2.76 (1.48–5.15); 0.001	5.44 (2.01–14.71); 0.001
Education ≥12 years	1	1
Lower financial status **	2.53 (1.56–4.12); <0.001	3.64 (1.51–8.76); 0.004
Higher financial status **	1	1
*Infertility treatment*		
Yes	1.65 (0.68–4.01); 0.269	4.29 (1.06–17.39); 0.041
No	1	1

* AOR-a: adjusted odds ratios (and 95% confidence intervals) of GH and PE calculated in multiple logistic regression in model-a; the results were adjusted for maternal age, pre-pregnancy BMI, primiparity, gestational weight gain (GWG) out of the range and smoking in the first trimester (*p*-value < 0.05 was assumed to be significant). ** Financial status was assessed on the 5-point Likert scale (Table 1): Lower financial status includes the levels 1–3; higher financial status includes the levels 4–5. GH: gestational hypertension; PE: preeclampsia. Controls: normotensive women.

**Table 4:** Unadjusted and adjusted odds ratios for the association between paternal and maternal hypertension on gestational hypertension (GH) and preeclampsia (PE) in the whole cohort.

Risk Factors/Hypertension in the Family	Cases/Controls	OR (95% CI); *p*	AOR-a (95% CI); *p* *	AOR-b (95% CI); *p* *
		GH Risk		
In the mother	31/137	1.90 (1.18–3.06); 0.008	1.51 (0.90–2.52); 0.117	1.34 (0.79–2.29); 0.279
In the father	33/135	2.06 (1.29–3.28); 0.002	1.98 (1.20–3.28); 0.008	1.88 (1.12–3.17); 0.017
In the mother or father	51/230	1.86 (1.24–2.8); 0.003	1.66 (1.07–2.55); 0.022	1.53 (0.98–2.39); 0.063
In the mother and father **	13/42	2.60 (1.32–5.13); 0.006	2.18 (1.04–4.58); 0.039	1.98 (0.91–4.30); 0.084
Ref ***	59/496	1	1	1
		**PE Risk**		
In the mother	11/137	3.98 (1.66–9.57); 0.002	3.26 (1.30–8.16); 0.012	3.09 (1.20–7.98); 0.020
In the father	5/135	1.84 (0.62–5.47); 0.274	1.60 (0.52–4.90); 0.412	1.40 (0.41–4.74); 0.592
In the mother or father	13/230	2.80 (1.21–6.49); 0.016	2.34 (0.99–5.55); 0.054	2.14 (0.87–5.29); 0.098
In the mother and father **	3/42	3.54 (0.94–13.37); 0.062	2.81 (0.68–11.53); 0.152	2.59 (0.58–11.56); 0.212
Ref ***	10/496	1	1	1

* AOR: adjusted odds ratios (and 95% confidence intervals) calculated in multiple logistic regression after adjusting for maternal age, pre-pregnancy body mass index (BMI), primiparity, gestational weight gain (GWG) out of the range and smoking in the first trimester (AOR-a), plus prior hypertension in pregnancy and infertility treatment (AOR-b) (*p*-value < 0.05 was assumed to be significant); ** In the mother and father simultaneously. *** Reference category: ‘Absence of hypertension in the family’ (in the parents and other relatives, i.e., sister(s), brother(s), grandmother(s) and grandfather(s)). Cases: GH i.e., gestational hypertension; PE i.e., preeclampsia; Controls: normotensive women. OR: unadjusted odds ratios.

**Table 5:** Adjusted odds ratios for the association between paternal and maternal hypertension on gestational hypertension (GH) and preeclampsia (PE) in the subgroups of BMI or smoking categories.

Risk Factors/Hypertension in the Parents	Cases/Controls	GH Risk	Cases/Controls	PE Risk
		AOR-a (95% CI); *p* *		AOR-a (95% CI); *p* *
Whole cohort				
In the mother	31/137	1.51 (0.90–2.52); 0.117	11/137	3.26 (1.30–8.16); 0.012
In the father	33/135	1.98 (1.20–3.28); 0.008	5/135	1.60 (0.52–4.9); 0.412
Ref **	59/496	1	10/496	1
Normal BMI				
In the mother	10/90	1.23 (0.56–2.69); 0.612	2/90	1.40 (0.26–7.60); 0.699
In the father	15/92	1.84 (0.92–3.67); 0.083	1/92	0.70 (0.08–6.34); 0.753
Ref **	29/351	1	5/351	1
Underweight				
In the mother	0/6	-	1/6	-
In the father	1/6	-	1/6	-
Ref **	0/30	1	1/30	1
Overweight				
In the mother	8/25	1.87 (0.69–5.11); 0.221	2/25	3.12 (0.37–26.42); 0.296
In the father	11/26	2.67 (1.02–7.02); 0.046	0/26	-
Ref **	14/87	1	2/87	1
Obesity				
In the mother	13/16	1.60 (0.59–4.35); 0.359	6/16	6.51 (1.05–40.25); 0.044
In the father	6/11	1.09 (0.31–3.83); 0.894	3/11	3.57 (0.48–26.5); 0.214
Ref **	16/28	1	2/28	1
Never smoked				
In the mother	23/104	1.87 (1.05–3.34); 0.034	10/104	5.31 (1.91–14.8); 0.001
In the father	21/114	1.70 (0.94–3.08); 0.079	4/114	1.99 (0.56–7.07); 0.289
Ref **	46/421	1	7/421	1
Smokers				
In the mother	8/33	0.60 (0.19–1.96); 0.400	1/33	0.88 (0.07–11.38); 0.921
In the father	12/21	3.15 (1.16–8.54); 0.024	1/21	1.73 (0.13–23.57); 0.682
Ref **	13/75	1	3/75	1
Smokers in the 1st tr.				
In the mother	2/8	0.39 (0.05–3.43); 0.398	1/8	2.03 (0.09–44.27); 0.653
In the father	7/5	4.71 (1.01–21.96); 0.048	1/5	6.32 (0.14–276.9); 0.339
Ref **	8/27	1	2/27	1

* AOR-a: adjusted odds ratios (and 95% confidence intervals) calculated in the multiple logistic regression (model-a) after adjusting for maternal age, pre-pregnancy body mass index (BMI), primiparity, gestational weight gain (GWG) out of the range and smoking in the first trimester (in the subgroups, BMI and smoking was excluded from the confounding variables mentioned) (*p*- value < 0.05 was assumed to be significant). ** Reference category: ‘Absence of hypertension in the family’ (in the parents and other relatives, i.e., sister(s), brother(s), grandmother(s) and grandfather(s)). Cases: GH i.e., gestational hypertension; PE i.e., preeclampsia; Controls: normotensive women.

## Data Availability

The data presented in this study are available on request from the corresponding author. The data are not publicly available as it contains a variety of patient information and covers a much wider range than needed for the analysis presented here.

## Supplementary Materials

The following are available online at https://www.mdpi.com/article/10.3390/ijerph18137045/s1, Table S1: The adjusted odds ratios of two forms of pregnancy-induced hypertension for basic risk factors (set of data), Table S2: Unadjusted and adjusted odds ratios for the association between paternal and maternal hypertension on gestational hypertension (GH) and preeclampsia (PE), compared to ‘Absence of hypertension in the parents’.

## Abbreviation

AOR	Adjusted odds ratio
BMI	Body mass index
CI	Confidence intervals
FIGO	International Federation of Gynecology and Obstetrics
GDM	Gestational diabetes mellitus
GH	Gestational hypertension
GWG	Gestational weight gain
OR	(Unadjusted) Odds ratio
PE	Preeclampsia
PIH	Pregnancy-induced hypertension
SOGC	Society of Obstetricians and Gynaecologists of Canada
WHO	World Health Organization
