# Supplementary material for: The Association of Familial Hypertension and Risk of Gestational Hypertension and Preeclampsia
Source: Int J Environ Res Public Health. 2021 Jul 1;18(13):7045. doi: 10.3390/ijerph18137045 (PMC8296897; doi:10.3390/ijerph18137045)
Supplement: Supplementary file 1 [file ijerph-18-07045-s001.zip › ijerph-1219328-supplementary.pdf]

International Journal of
Environmental Research and Public Health

MDPI

Supplementary Table S1. Unadjusted and adjusted odds ratios for the association between basic risk factors on gestational hypertension (GH) and preeclampsia (PE) (set of data).

Risk factors	Cases/Controls	OR (95% CI), *p*	AOR-a (95% CI), *p*
		GH risk	
Maternal age (years):			
≥40	16/59	3.23 (1.41−7.38); 0.005	2.97 (1.2−7.32) 0.018
Age 18−24	2/36	0.66 (0.14−3.12); 0.602	0.51 (0.1−2.53) 0.413
25−29 years	11/131	1	1
Pre-pregnancy BMI (kg/m²):			
Obesity (≥30)	31/58	5.60 (3.32−9.43); <0.001	4.72 (2.73−8.14) <0.001
Overweight (25−29.9)	30/139	2.26 (1.39−3.68); 0.001	2.04 (1.23−3.37) 0.005
Underweight (<18.5)	1/44	0.24 (0.03−1.76); 0.160	0.23 (0.03−1.69); 0.148
Normal BMI (18.5−24.9)	51/534	1	1
GWG above the range	62/263	2.45 (1.53−3.92); < 0.001	1.9 (1.15−3.12); 0.012
GWG in the range	29/301	1	1
GWG below the range	22/211	1.08 (0.61−1.94); 0.790	1.09 (0.59−2.01); 0.779
Smoking in the 1st tr.	17/37	3.60 (1.94−6.68); <0.001	3.51 (1.79−6.89); <0.001
Smoking before pregnancy	31/133	1.83 (1.16−2.87); 0.009	1.64 (1.01−2.66); 0.044
Never smoked	82/642	1	1
Prior GH/PE	12/4	22.9 (7.25−72.36); <0.001	37.98 (11.16−129.26); <0.001
No prior GH/PE	101/771	1	1
Primiparity	53/318	1.27 (0.85−1.89); 0.238	1.90 (1.22−2.96); 0.005
Multiparity	60/457	1	1
Infertility treatment	8/29	1.96 (0.87−4.4); 0.103	1.65 (0.68−4.01); 0.269
No Infertility treatment	105/746	1	1
		PE risk	
Maternal age (years):			
≥40	1/59	1.11 (0.1−12.49); 0.933	0.94 (0.08−11.61); 0.963
25−29	2/131	1	1
18−24	2/36	3.64 (0.5−26.74); 0.204	3.26 (0.42−25.04); 0.256
Pre-pregnancy BMI (kg/m²):			
Obesity (≥30)	9/58	9.21 (3.52−24.11); < 0.001	8.68 (3.25−23.18); <0.001
Overweight (25−29.9)	4/139	1.71 (0.52−5.63); 0.379	1.68 (0.5−5.63); 0.397
Underweight (<18.5)	2/44	2.70 (0.57−12.87); 0.213	2.56 (0.52−12.62); 0.248
Normal BMI (18.5−24.9)	9/534	1	1
GWG above the range	11/263	1.57 (0.62−3.97); 0.337	1.15 (0.44−3); 0.770
GWG in the range	8/301	1	1
GWG below the range	5/211	0.89 (0.29−2.76); 0.842	0.84 (0.26−2.67); 0.768
Smoking in the 1st tr.	3/37	2.60 (0.74−9.16); 0.136	2.79 (0.76−10.27); 0.124
Smoking before pregnancy	4/133	0.97 (0.33−2.87); 0.950	0.88 (0.29−2.65); 0.817
Never smoked	20/642	1	1
Prior GH/PE	3/4	27.54 (5.8−130.83); <0.001	31.11 (5.83−165.98); <0.001
No prior GH/PE	21/771	1	1
Primiparity	11/318	1.22 (0.54−2.75); 0.638	1.55 (0.64−3.77); 0.332
Multiparity	13/457	1	1
Infertility treatment	3/29	3.68 (1.04−13.03); 0.044	4.29 (1.06−17.39); 0.041
No infertility treatment	21/746	1	1

* AOR-a: adjusted odds ratios (and 95% confidence intervals) of GH and PE calculated in multiple logistic regression in model-a; the results were adjusted for maternal age, pre-pregnancy BMI, primiparity, gestational weight gain (GWG) out of the range and smoking in the first trimester (p-value < 0.05 was assumed to be significant). GH: gestational hypertension; PE: preeclampsia. Controls: normotensive women.

Supplementary Table S2. Unadjusted and adjusted odds ratios for the association between paternal and maternal hypertension on gestational hypertension (GH) and preeclampsia (PE), compared to 'Absence of hypertension in the parents'

Risk factors/Hypertension in the parents	Cases/Controls	OR (95% CI); *p*	AOR-a (95% CI); *p* *	AOR-b (95% CI); *p* *
**GH risk**				
In the mother	31/137	2.01 (1.25–3.21); 0.004	1.61 (0.97–2.67); 0.065	1.46 (0.86–2.48); 0.165
In the father	33/135	2.17 (1.36–3.45); 0.001	2.09 (1.27–3.44); 0.004	2.03 (1.21–3.42); 0.008
In the mother or father	51/230	1.97 (1.31–2.94); 0.001	1.75 (1.14–2.69); 0.010	1.65 (1.06–2.58); 0.028
In the mother and father **	13/42	2.75 (1.40–5.40); 0.003	2.31 (1.11–4.82); 0.025	2.14 (0.98–4.65); 0.056
Ref ***	61/541	1	1	1
**PE risk**				
In the mother	11/137	3.95 (1.68–9.30); 0.002	3.28 (1.34–8.04); 0.009	3.12 (1.24–7.85); 0.016
In the father	5/135	1.82 (0.62–5.33); 0.274	1.58 (0.52–4.76); 0.416	1.37 (0.42–4.53); 0.605
In the mother or father	13/230	2.78 (1.23–6.30); 0.014	2.35 (1.02–5.45); 0.046	2.15 (0.89–5.17); 0.088
In the mother and father **	3/42	3.51 (0.94–13.08); 0.061	2.78 (0.69–11.24); 0.151	2.57 (0.59–11.2); 0.207
Ref ***	11/541	1	1	1

* AOR: adjusted odds ratios (and 95% confidence intervals) calculated in multiple logistic regression after adjusted for maternal age, pre-pregnancy BMI, primiparity, gestational weight gai (GWG) out of the range and smoking in the first trimester (AOR-a) plus prior hypertension in pregnancy and infertility treatment (AOR-b) (*p*-value < 0.05 was assumed to be significant); ** In the mother and father simultaneously; *** Reference category: 'Absence of hypertension in the parents'. Cases: GH i.e. gestational hypertension; PE i.e. preeclampsia; Controls: normotensive women.
